# Topics in Burn Critical Care—Chest Pain

**Published:** 2013-03-15

**Authors:** Neeraj M. Shah, Justin Klaff, Stephen M. Milner, Leigh A. Price, Kevin B. Gerold

**Affiliations:** ^a^King's College London Medical School, London, UK; ^b^Johns Hopkins Burn Center, Johns Hopkins University School of Medicine, Baltimore, MD

**Figure:**
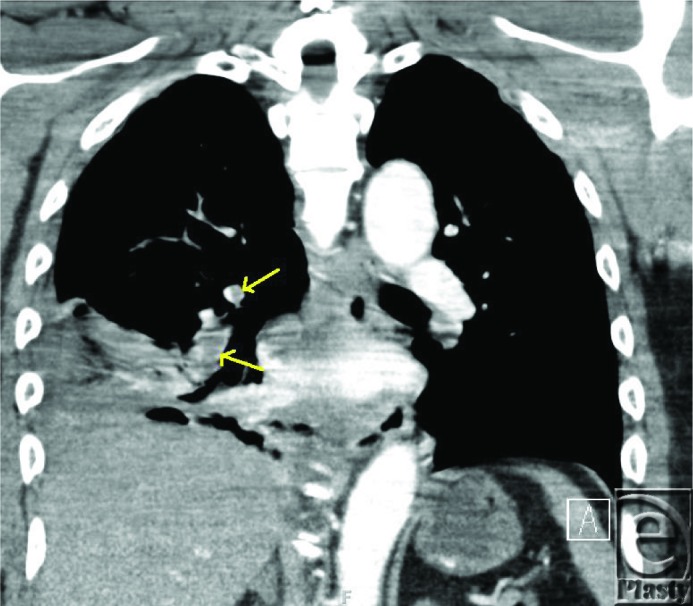


## DESCRIPTION

A 57-year-old gentleman sustained third-degree burns to his right arm and left leg following a high-voltage electrical injury. On hospital day 23, he reported an acute onset of sharp, right-sided chest pain exacerbated during inspiration. On examination, he was tachypneic.

## QUESTIONS

1. **What is the differential diagnosis for the patient's chest pain?**
2. **What are the risk factors associated with pulmonary embolism (PE) in burn patients?**
3. **How do we diagnose and manage burn patients with PE?**
4. **What regimens are currently recommended for prophylaxis of venous thromboembolism in burn patients?**

## DISCUSSION

Pulmonary complications will occur in 16% to 41% of patients with thermal injury.^1^ The differential diagnosis for acute chest pain includes PE, pneumonia, myocardial infarction, and pneumothorax. Pleuritic chest pain, dyspnea, hemoptysis, pleural friction rub, and desaturation are characteristic diagnostic features for PE. Initial screening investigations include chest radiography, electrocardiogram, and arterial blood gas. Common findings on chest radiography include atelectasis or areas of increased opacity, although these are nonspecific. An arterial blood gas has limited usefulness, but it may show mild hyperventilation and hypoxemia, and an increased A-a gradient. An electrocardiogram will most commonly reveal sinus tachycardia and occasionally the characteristic “S1Q3T3” sign (S-wave in lead I, Q-wave and inverted T-wave in lead III). When a PE is suspected in a seriously injured burn patient, spiral (helical) computerized tomography (CT) or ventilation-perfusion scan are the most useful imaging modalities. Ventilation-perfusion scanning provides less radiation, but is less diagnostic in patients with preexisting lung disease or an abnormal chest radiograph. Presently, spiral CT has replaced pulmonary arteriogram as the standard of care means to diagnose PE.^2^

A recent analysis of the American Burn Association's National Burns Repository reported the incidence of venous thromboembolism and PE in burn patients as 1.2% and 0.18%, respectively.^3^ In patients with electrical injury who underwent early fasciotomy, the incidence of deep vein thrombosis was as high as 7.55%.^4^ Risk factors include hypercoagulability, impaired endothelial integrity, and immobilization (Virchow's Triad); inevitably, all present in burn patients. Thermal injury triggers a systemic inflammatory response, which activates adaptive mechanisms that reduce levels of antithrombin III and protein C and S.^5^^,^^6^ This results in a hypercoagulable state. The thermal injury also results in a direct injury to vascular endothelium, and the care of burn wounds often requires extended periods of immobilization also increasing the risk for venous thrombus formation. Additional independent risk factors for PE in burn patients include total body surface area burned (proportional relationship), length of stay in intensive care unit, number of operations, inhalation injury,^3^ and presence of a central venous catheter.^7^

In hemodynamically stable patients, treatment involves anticoagulation therapy using low-molecular-weight heparin (LMWH), unfractionated heparin, or fondaparinux. A meta-analysis demonstrated that LMWH is as effective as unfractionated heparin.^8^ There are no protocols specific for the treatment of burn patients. The American College of Chest Physicians currently recommends treating acute PE initially with LMWH and warfarin (Coumadin). LMWH should be continued until the international normalized ratio (INR) is greater than 2.0 for 24 hours.^9^

In hemodynamically unstable patients, thrombolysis (mechanical or pharmacologic) should be considered.^10^ Emergency embolectomy may be life-saving in patients with massive PE. Patients with a PE remain at continued risk of further thromboembolic events, thus needing long-term anticoagulation, usually with Vitamin K antagonists.

The duration of anticoagulation is determined by the risk of recurrence. The cause of PE in burn patients without other risk factors is likely the result of systemic inflammation, which subsides with time. These patients are candidates for the limited 3- to 6-month treatment protocol, maintaining a target INR of 2.0 to 3.0. Patients with additional risk factors are likely to require indefinite treatment.

While there are no randomized controlled trials on thromboprophylaxis in burn patients, these patients are at an increased risk of deep vein thrombosis and PE, so prophylaxis is essential. The American College of Chest Physicians' guidelines on venous thromboembolism prophylaxis currently recommends the use of routine thromboprophylaxis.^11^ Busche et al^12^ report that LMWH had decreased rates of deep vein thrombosis and heparin-induced thrombocytopenia, when compared with unfractionated heparin. Lin et al^13^ suggests a higher initial dose of enoxaparin in burns patients because these patients have lower initial levels of antifactor Xa. The regression analysis of this study showed that the initial dose of unfractionated heparin is 36 mg every 12 hours.^13^
